# Innovations in Bioactive Materials for Dental Pulp Vitality Preservation in Children and Adolescents

**DOI:** 10.3390/app15094699

**Published:** 2025-04-24

**Authors:** Purva K. Desai, Shreya M. Hiwalkar, Hyun-Joo Kim, Jonghyun Shin, Hyo-Seol Lee, Ho-Wook Jun, Kyounga Cheon

**Affiliations:** 1 Department of Pediatric Dentistry, School of Dentistry, University of Alabama at Birmingham, Birmingham, AL 35294, USA;; 2 Department of Biomedical Engineering, School of Engineering, University of Alabama at Birmingham, Birmingham, AL 35294, USA;; 3 Department of Periodontology, Dental and Life Science Institute, School of Dentistry, Pusan National University, Yangsan 50612, Republic of Korea;; 4 Department of Pediatric Dentistry and Dental and Life Science Institute, School of Dentistry, Pusan National University, Yangsan 50612, Republic of Korea;; 5 Department of Pediatric Dentistry, School of Dentistry, Kyung Hee University, Seoul 02447, Republic of Korea;

**Keywords:** bioactive materials, vital pulp therapy, pulp regeneration, tissue engineering

## Abstract

Preserving dental pulp vitality is crucial in pediatric and adolescent dentistry to promote long-term oral health and reduce the need for invasive procedures. Vital pulp therapy (VPT) enhances pulp healing and dentin formation through advanced pulp capping materials. While calcium hydroxide-based materials laid the foundation for VPT, calcium silicate-based materials like mineral trioxide aggregate, Biodentine, and TheraCal offer superior biocompatibility and sealing properties. Recent advancements focus on regenerative strategies that enhance biocompatibility, antibacterial efficacy, and anti-inflammatory effects. Tissue engineering approaches, including stem cells, growth factors, and peptide-based scaffolds, are being explored to improve pulp regeneration and long-term treatment success. This review highlights recent developments in VPT for pediatric and adolescent patients, emphasizing minimally invasive techniques, clinical challenges, and the potential of emerging biomaterials. Continued research into biomaterial efficacy and regenerative capabilities holds promise for advancing VPT, ensuring more effective and biologically driven treatment strategies for young patients.

## 1. Introduction

Preserving dental pulp vitality is paramount to the success of vital pulp therapy (VPT) and long-term oral health care. The dental pulp plays a crucial role in maintaining tooth vitality, providing essential nutrients, and responding to injuries through reparative processes [1]. The loss of pulp vitality necessitates more invasive treatment options, such as root canal therapy, extractions, implants, or dentures, which can be costly and time-consuming. Therefore, strategies aimed at maintaining the health of all or part of the pulp are essential for extending tooth longevity and minimizing the need for invasive interventions.

Early diagnosis and timely treatment are critical in preserving dental pulp vitality. Regular dental check-ups starting at an early age help prevent and identify oral health issues early. The American Academy of Pediatric Dentistry (AAPD) recommends that children have their first dental visit by their first birthday or within six months after their first tooth appears. This early start, often called establishing a “dental home”, allows dentists to monitor tooth development, provide preventive care, and educate parents on proper oral hygiene for infants and toddlers [2].

Vital pulp therapy (VPT) involves a continuum of procedures, ranging from sealing the unexposed pulp to disinfecting and stabilizing the exposed pulp using bioactive materials. The success of VPT relies heavily on the selection and performance of pulp capping materials. Traditional materials, such as calcium hydroxide (CH), have historically been the cornerstone of VPT due to their ability to stimulate mineralized tissue formation and provide antibacterial effects through their high alkalinity. Despite these advantages, CH-based materials present significant limitations that impact long-term clinical success. Notably, they exhibit high solubility in oral fluids, which compromises their durability and can lead to early dissolution. Their poor sealing ability also increases the risk of microleakage and bacterial infiltration, undermining the treatment outcome. Furthermore, CH has weak mechanical properties, leading to structural instability under functional loads. These deficiencies have driven the search for alternative bioactive materials that offer superior biocompatibility, sealing ability, and mechanical strength, thereby enhancing pulp preservation and regenerative outcomes [3–5]. This evolving need underscores the development and clinical integration of new-generation materials such as calcium silicate cements, bioceramics, and biomimetic scaffolds.

Recent advancements in bioactive materials have markedly enhanced the clinical outcomes of VPT. Among these, calcium silicate-based materials such as mineral trioxide aggregate (MTA), Biodentine, and TheraCal LC have demonstrated superior biocompatibility, sealing ability, and mechanical integrity compared to traditional materials like calcium hydroxide. MTA is known for its ability to promote dentin bridge formation and pulp healing due to the sustained release of calcium ions and the induction of an alkaline environment conducive to tissue regeneration [6–8]. However, its prolonged setting time, potential for discoloration, and relatively high cost can limit its use in certain clinical settings [9–11]. Biodentine, another tricalcium silicate cement, offers a shorter setting time and improved handling characteristics compared to MTA, with favorable biological responses such as odontoblastic differentiation and low cytotoxicity [12]. Nonetheless, Biodentine exhibits lower radiopacity and wash-out resistance than MTA, which may affect its performance in long-term or complex cases [13]. TheraCal LC, a light-curable resin-modified calcium silicate material, is appreciated for its ease of application and immediate setting under light exposure. It releases calcium ions and can promote pulp healing in shallow exposures. However, studies have raised concerns about the cytotoxicity of its resin components (e.g., BisGMA, HEMA, and TEGDMA), which may provoke inflammatory responses in deeper pulp tissues [14].

Beyond these cements, regenerative strategies involving tissue engineering approaches—including stem cells, growth factors (e.g., TGF-β, VEGF, PDGF), and peptide- or protein-based scaffolds—are gaining momentum. These biomimetic approaches aim to go beyond passive sealing and promote true pulp-dentin complex regeneration. Materials such as platelet-rich plasma (PRP), chitosan, gelatin methacrylate (GelMA), and nitric oxide-releasing nanomatrix have demonstrated promising potential in promoting angiogenesis, modulating inflammation, and stimulating the recruitment and differentiation of dental pulp stem cells [15–17]. Despite their promise, many of these innovations remain in preclinical or early clinical phases, and challenges such as cost, scalability, regulatory approval, and long-term efficacy must still be addressed before widespread clinical adoption.

Collectively, these advances reinforce the clinical shift toward minimally invasive, biologically based approaches in VPT. The integration of regenerative biomaterials and diagnostic technologies supports long-term preservation of pulp vitality, enhances healing outcomes, and reduces the need for more invasive procedures, ultimately benefiting both pediatric patients and the dental profession.

To provide a comprehensive foundation for these advancements, it is essential to examine the diagnostic strategies and therapeutic indications that inform VPT decisions. This narrative review focuses on materials used in VPT for dental pulp capping, exploring their strengths, weaknesses, and clinical limitations. By analyzing the recent advances on both traditional and regenerative materials, this review provides insight into emerging clinical challenges and opportunities for innovation. A deeper understanding of pulp vitality, therapeutic mechanisms, and biomaterial design offers promising avenues for improving treatment efficacy and long-term oral health. Such advancements aim to improve clinical outcomes, particularly in preserving pulp vitality.

## 2. Literature Search Strategy

A comprehensive literature search was conducted using PubMed (Medline), Google Scholar, Scopus, Web of Science, and ScienceDirect. Keywords included “Indirect pulp treatment (IPT)”, “Direct pulp capping (DPC)”, “Pulpotomy”, “Vital Pulp Therapy”, “Tooth Vitality”, “Pulp Preservation”, “Pulp Regeneration”, “Wound Healing”, “Biocompatibility”, “Bioactive Dental Materials”, “Regenerative Dental Materials”, “Biological and Clinical Outcomes”, “Tissue Engineering”, “Minimally Invasive Dentistry”, and “Prevention”. Articles published in English peer-reviewed journals between January 1990 and December 2024 were included to ensure a thorough historical and contemporary scope for this review (Figure S1). Studies focusing exclusively on non-vital pulp treatments, such as root canals, pulpectomy, extractions, prosthetics, and implants, were excluded from the review.

## 3. Criteria of Vital Pulp Therapy

### 3.1. Tooth Structure and Caries Invasion

Understanding the tooth structure is essential for evaluating dental pulp capping methods, as each layer, enamel, dentin, cementum, pulp, and alveolar bone, plays a specific role in tooth health and response to injury. Enamel is the outermost layer, which protects the dentin and also aids in mastication. Dentin is the second layer, which supports the enamel and also protects the pulp with growth factors as a defense. The cementum covers the dentin and also connects the tooth to the alveolar bone [18]. The pulp, the innermost layer, houses blood vessels, nerves, and connective tissue [19]. The alveolar bone supports teeth with compact and cancellous bone and a cribriform plate [20].

Dental caries is a chronic and multifaceted condition that is characterized by bacterial invasion, traumatic injuries, and loss of enamel and dentin structure. It is a pathological process that is spread through an inflammatory response, leading to exposure of enamel, dentin, and the pulp chamber, resulting in pulpitis, periapical abscess, and periodontitis with bone loss. Caries lesions are initiated by undisturbed dental plaque accumulation and biochemical bacterial biofilm metabolism, which utilizes dietary sugars to grow and ferment the lactic acid process [21]. In the early stages, the pulpal reaction is reversible; however, as caries progress, the formation of tertiary dentin is accompanied by increased nociceptive sensitivity due to the activation of pulpal nerve fibers and inflammatory mediators. Tertiary dentin can be observed as two dentin structures. One is reactionary dentin (formed due to a mild stimulus by existing odontoblasts) [22], and the other is reparative dentin (formed due to deep caries close to the pulp by odontoblast-like cells without dentinal tubules) [23]. The current biomedical approach aims to replace damaged dental tissue using innovative biomaterials such as calcium silicate-based cement, bioactive glass, and hydrogels by controlled ion release and enhanced mechanical properties to optimize pulp tissue regeneration and dentin bridge formation. These innovations aim to improve the material’s sealing ability, antibacterial effects, and long-term clinical outcomes.

### 3.2. Diagnosis and Treatment Planning

To determine appropriate treatment options for pulp vitality and necrosis, a comprehensive diagnosis, including pain history, clinical examination, radiographic imaging, three-dimensional cone-beam computed tomography (CBCT), and pulp vitality tests, is of utmost importance [24]. Clinical examination should be done after dental plaque has been removed, the surface is dry and is examined under proper lighting. Additional caries detection methods like Quantitative Light-induced Fluorescence, DIAGNOdent pen, Fiber-optic Transillumination, and Electrical Conductance can also be used. These methods classify cavities based on severity and activity, often determined by reflection and texture [25,26]. A radiographic examination should also be rendered to see the extent of the caries spread and determine the stage of the carious spread [25–27]. Another important aspect of the diagnosis includes the differentiation between the affected and infected dentin. The infected dentin is the part of the tooth that will feel soft and is demineralized; on the other hand, the affected dentin is the part of the tooth that is stained, but still has retained hardness and integrity [28]. Recent studies have highlighted the importance of advanced diagnostic tools such as CBCT in providing detailed three-dimensional images, which help in precise diagnosis and treatment planning [29]. CBCT has been shown to improve the accuracy of detecting periapical lesions and assessing the extent of pulp involvement. Additionally, sensitivity tests, including electric pulp testing and thermal testing, are crucial in evaluating the responsiveness of the pulp to stimuli, aiding in the diagnosis of pulp vitality.

The treatment options for pulpal disease vary based on factors like the tooth’s strategic importance, restorative prognosis, patient preference, and overall oral health [30]. The appropriate treatment options can range from VPT to tooth extraction and extensive prosthetic restorations. Learning from consequential evidence, maintaining tooth vitality in the early stages is crucial for tooth longevity and oral health. Early and minimal interventions using pulp capping procedures would deliver optimal disinfection and facilitate the innate healing response of the root canal system of primary teeth, which can be succeeded by the permanent tooth without introducing a periapical or periodontal pathology. The various methods in vital pulp treatment include IPT, DPC, partial pulpotomy, and full pulpotomy. A pulpectomy is used in non-vital cases, but surgical interventions like extraction may be necessary in beyond-restorable cases [31]. Among these intervention approaches, DPC could maintain pulpal vitality while promoting natural reparative processes at the beginning of the caries progression, making it a key approach in modern minimally invasive dentistry.

### 3.3. Vital Pulp Therapy

Vital pulp therapy (VPT) aims to maintain the health and function of the dental pulp following injury or carious exposure. The 2024 AAPD Clinical Practice Guidelines emphasize that VPT is indicated for both primary teeth with a vital pulp and no signs of radicular pathology, and for immature permanent teeth with vital pulp, regardless of whether pulpitis is reversible or in early stages of irreversible inflammation [31,32].

**Figure 1.**
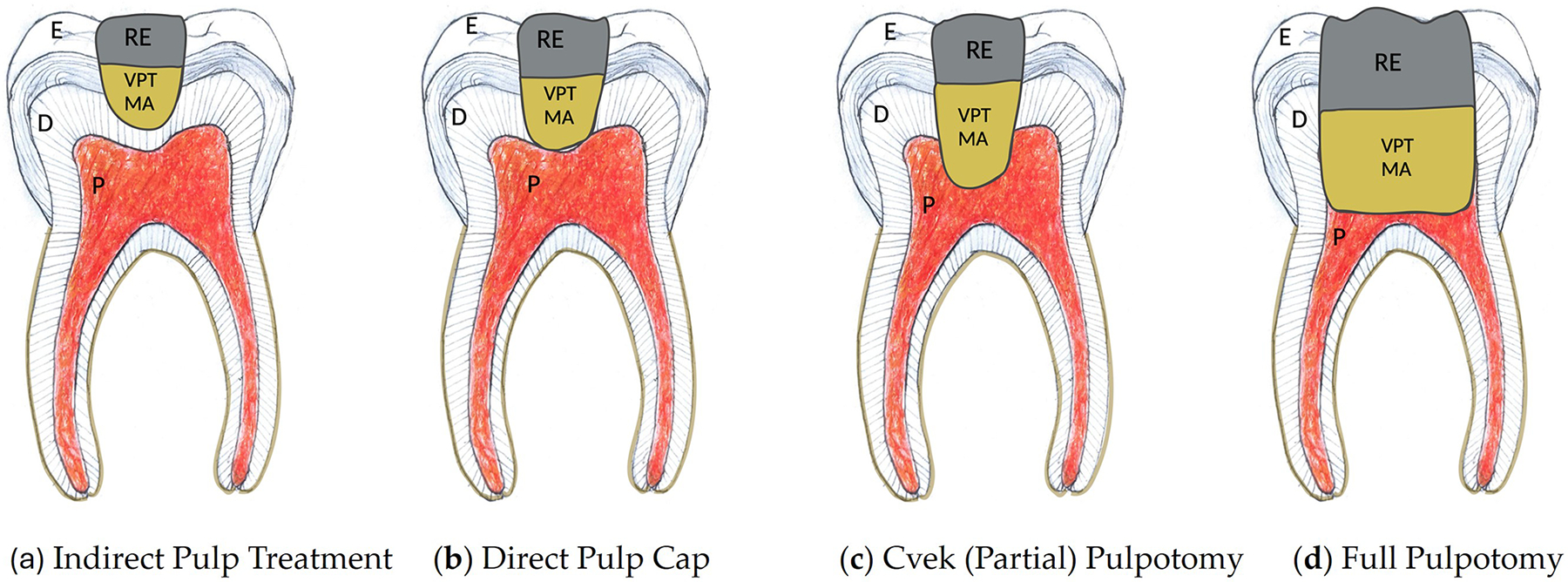
Vital Pulp Therapy Techniques (original illustration by the authors): (**a**) Indirect Pulp Treatment: A biocompatible capping material is placed over remaining demineralized but non-infected dentin to avoid pulp exposure; (**b**) Direct Pulp Cap: The capping material is applied directly to a small, exposed pulp area due to mechanical or traumatic exposure; (**c**) Cvek (Partial) Pulpotomy: A portion of the inflamed coronal pulp is removed to preserve healthy radicular pulp. A capping material is applied to the prepared pulp exposure; (**d**) Full Pulpotomy: The entire coronal pulp is removed, and the radicular pulp is covered with a pulp capping material and sealed. E. Enamel, D. Dentin, P. Pulp, RE. Restoration, VPT MA. Vital Pulp Therapy Material.

Indirect pulp treatment (IPT) is a method used to manage deep carious lesions near the pulp without direct pulp exposure. This approach leaves a layer of affected, but not infected, dentin to avoid pulpal exposure and places a biocompatible material—often a calcium hydroxide or calcium silicate-based liner—over it [4]. The goal is to arrest caries progression, preserve pulp vitality, and promote reparative dentin formation without repeated caries excavation (Figure 1a).

Direct pulp capping (DPC) is the application of a biocompatible material directly onto a small, exposed pulp site, typically due to trauma or iatrogenic injury. According to the AAPD, DPC may be considered for primary teeth only in mechanical or traumatic exposures with normal pulp and no symptoms, and for immature permanent teeth with small carious or mechanical exposures when bleeding is controlled [31,33]. The procedure relies on an effective coronal seal and the regenerative potential of the selected material to promote dentin bridge formation and preserve pulp vitality [4,31] (Figure 1b).

Pulpotomy involves removing the inflamed coronal pulp while preserving the vital radicular pulp. In primary teeth, full pulpotomy is recommended as the treatment of choice in cases of carious pulp exposure with vital pulp and controlled bleeding. The AAPD recommends bioactive materials like MTA and Biodentine over formocresol due to superior success rates and favorable biological response. In permanent teeth with immature roots, partial pulpotomy (Cvek pulpotomy) is indicated when exposure is recent, and bleeding is controlled, allowing the remaining pulp to facilitate root development and apexogenesis [34–36]. Partial pulpotomy (Figure 1c) removes 2–3 mm of inflamed tissue beneath the exposure site, preserving deeper, healthy pulp in immature permanent teeth. Partial pulpotomy is advantageous over full pulpotomy in the preservation of cell-rich coronal pulp tissue, allowing dentin apposition and the ability to perform pulp testing [37,38]. The partial pulpotomy has a 98% success rate in reversible pulpitis cases and a 75% success rate in irreversible pulpitis cases after one year [35]. Success rates remain high (>90%) when appropriately diagnosed and managed, even in cases previously classified as irreversible pulpitis. A full pulpotomy (Figure 1d) removes all coronal pulp tissue from the pulp chamber and places bioactive material, leaving healthy radicular pulp with the indication of reversible pulpitis or after a traumatic pulp exposure without evidence of radiographic signs of infection or pathologic resorption [31]. It is considered appropriate for both primary molars and immature permanent teeth when pulp vitality can be confirmed, and bleeding is controlled within 5–10 min. This procedure is commonly used in primary molars with extensive caries [39–41] and it has been considered a treatment choice for exposed permanent teeth with mild symptoms or irreversible pulpitis [42]. In a systematic review, pulpotomy was performed on teeth with irreversible pulp without any other symptoms, and had a clinical success rate of 97.4% at one year and 93.97% at three years [43].

The success of pulpotomy depends on factors such as accurate pulpal diagnosis, caries removal, hemostasis, material selection, and final restoration integrity [44]. The AAPD guidelines stress the importance of using high-quality coronal restorations to prevent microleakage and ensure the long-term success of VPT.

## 4. Dental Biomaterials for VPT

VPT using IPT, DPC, or pulpotomy has evolved with various dental biomaterials over the decades (Table 1). The traditional “Gold standard”, calcium hydroxide (CH), demonstrated antibacterial effects due to its alkaline pH, OH^−^ ion release, and localized necrosis, stimulating calcium bridge formation [3,5]. However, CH has been criticized for its high solubility, low sealing capacity, weak physical properties, and unpredictable clinical success [3–5].

Bioceramic materials using calcium silicate cement (CSC), including mineral trioxide aggregate (MTA), Biodentine, and Thera Cal LC, have shown a higher overall success rate over ten years [4,45–47]. Recent developments in CSCs offer benefits like improved flowability and being dispensed in bulk [48]. The field’s dedication to preserving natural dentition and minimally invasive approaches has resulted in improved biocompatibility and use, resulting in improved overall patient outcomes [49]. Continuing research and innovation in this area are essential to furthering the effectiveness and predictability of dental pulp capping, aligning with evidence-based practice and patient-centered care. Table 1 provides an organized overview of dental biomaterials that have been used in VPT.

### 4.1. Restorative Dentistry and Pulp Preservation

As restorative dentistry has progressed, materials with enhanced biological and mechanical properties have emerged. Although traditional glass ionomer cements (GICs) and resin-modified glass ionomer cements (RMGICs) are not primary agents for direct pulp capping, their contributions to pulp vitality preservation through secondary sealing effects are increasingly recognized. GICs are appreciated for their fluoride release, chemical adhesion to tooth structure, and biocompatibility. However, they are limited in high-stress applications due to low fracture toughness and poor damage tolerance limitation supported by recent studies exploring their atomic and vibrational structure during setting [50]. RMGICs were introduced to improve mechanical resilience by combining resin components with GICs, but they still face challenges such as insufficient sealing in deep cavities and risks of bacterial microleakage, which can lead to pulp inflammation.

These limitations underscore the need for next-generation DPC materials that offer robust sealing, intrinsic antibacterial properties, and superior mechanical characteristics. Furthermore, the long-term success of VPT depends not only on biologically favorable pulp capping agents but also on the definitive coronal restoration. A well-sealed durable restoration is essential to prevent microleakage and maintain the therapeutic effects of pulp preserving interventions. Advances in biomimetic restorative materials may substantially enhance clinical outcomes by providing both bioactivity and mechanical resilience, ultimately supporting pulp vitality and reducing failure rates [56,57].

Building on these restorative and material innovations, the following section explores regenerative biomaterials and emerging biotechnologies that further enhance VPT outcomes.

### 4.2. Recent Advancements in the Vital Pulp Therapy (Table 2)

Recent research has highlighted the potential of biocompatible materials such as Collagen, Chitosan, Alginate, platelet-rich plasma (PRP), and fibrin (PRF), and decellularized human teeth, for pulp preservation. These materials offer improved sealing, biocompatibility, and dentin regeneration capabilities. Additionally, emerging techniques like laser therapy and nitric oxide-releasing nanomatrix show promise in maintaining pulp vitality and promoting dentin formation [58]. These advancements represent a shift towards biologically driven approaches in VPT, aiming to optimize outcomes by harnessing the natural healing potential of the pulp-dentin complex.

#### 4.2.1. Platelet-Rich Plasma/Fibrin, Chitosan, and Collagen

Platelet-rich plasma (PRP) and platelet-rich fibrin (PRF), derived from a patient’s autologous blood, are rich sources of bioactive molecules that stimulate tissue regeneration and dentin bridge formation. These preparations contain high concentrations of growth factors, including vascular endothelial growth factor (VEGF), transforming growth factor-beta (TGF-β), platelet-derived growth factor (PDGF), insulin-like growth factor (IGF), and epidermal growth factor (EGF) [67,68]. These factors play crucial roles in cell recruitment, angiogenesis, odontoblastic differentiation, and modulation of the inflammatory response, making PRP/PRF highly promising adjuncts for direct pulp capping. Its anti-inflammatory properties can also reduce tissue inflammation and accelerate healing. However, further research is needed to optimize its clinical application and long-term effects [62,63,69].

Chitosan-based scaffolds, particularly those functionalized with calcium silicate or silver-doped bioactive glass, offer dual benefits of bioactivity and antimicrobial action. They support odontogenic differentiation and have been shown to stimulate VEGF release, enhance cell proliferation, and promote dentin-pulp complex regeneration [15,61]. Similarly, gelatin-based systems, such as chitosan-gelatin blends and gelatin methacryloyl (GelMA) hydrogels, enhance cell adhesion, mimic extracellular matrix structure, and release endogenous factors to promote angiogenesis and reparative dentinogenesis [60,64].

Collagen-based systems, often reinforced with chitosan or crosslinked for stability, further contribute to regenerative pulp therapy by mimicking natural extracellular matrix environments, promoting angiogenesis and odontoblast differentiation [59]. These systems support cell migration and the delivery of signaling molecules necessary for pulp repair and revascularization. Together, these advanced materials create a microenvironment favorable for healing, reduce the need for extensive pulp removal, and enable the development of targeted, biologically driven treatment strategies.

#### 4.2.2. Alginate and Decellularized Human Teeth as Scaffolds for Tooth Regeneration

The article focuses on a novel approach to tooth regeneration using decellularized human teeth as scaffolds. This approach aims to engineer new tooth tissues by seeding these scaffolds with stem cells, specifically dental pulp stem cells and periodontal ligament stem cells. The goal is to create a biocompatible environment that promotes cell growth, differentiation, and tissue formation. This could potentially lead to the regeneration of damaged tooth structures, such as the pulp and periodontal ligament. The article delves into the details of the decellularization process, cell seeding, and in vivo transplantation studies. It discusses the potential of this technique to revolutionize dental regeneration and offers a promising avenue for future research in this field [52]. Zhang et al. developed injectable RGD-alginate/laponite hydrogel microspheres that co-encapsulate dental pulp stem cells and VEGF, demonstrating sustained growth factor release, high cell viability, and enhanced pulp-like tissue regeneration [16].

#### 4.2.3. Laser Therapy in Vital Pulp Therapy

Laser therapy has gained attention as a potential adjunct to traditional VPT techniques. Lasers, such as CO_2_, erbium (e.g., Er: YAG, Er, and Cr: YSGG), and diode lasers, each with different wavelengths and clinical applications, can exert diverse biological effects on pulp tissue. These effects include bacterial reduction, coagulation and hemostasis, enhanced cellular proliferation, and stimulation of dentin bridge formation [51,66]. However, reported clinical outcomes have been variable due to several factors, including differences in laser type and power settings, exposure duration, tissue penetration depth, operator technique, and case selection. Randomized controlled trials (RCTs) have demonstrated that laser-assisted VPT can result in reduced postoperative pain, accelerated healing, and improved clinical success rates in some cases. Yet, other studies have reported no significant difference or inconsistent histological outcomes compared to conventional treatments.

This variability underscores the need for standardized protocols and practitioner training. Furthermore, high-quality long-term studies are necessary to determine whether laser application consistently enhances clinical outcomes or if benefits are technique- and context-dependent [66]. Clinicians should weigh the potential benefits of laser disinfection and biological stimulation against practical considerations such as equipment cost, training requirements, and treatment complexity. Until more conclusive evidence is available, lasers may be best considered as an adjunct to, rather than a replacement for established VPT modalities.

#### 4.2.4. Nitric Oxide-Releasing Nanomatrix Gel

Recent research has explored the potential of nitric oxide (NO) releasing nanomatrix gels for pulp regeneration [70,71]. This biocompatible gel is designed to deliver antibiotics, NO, and potentially growth factors to damaged tooth pulp [72]. Studies have shown that this gel can promote pulp regeneration, including blood vessel formation and root thickening. While promising, further research is needed to confirm its effectiveness and optimize its use in clinical settings [17]. To further explore this potential, a study evaluated the efficacy of a doxycycline-loaded nitric oxide-releasing nano matrix gel in promoting pulp regeneration after replanting avulsed rat teeth. Rat molars were extracted and stored for varying durations before being replanted with or without the gel treatment. After eight weeks, the treated teeth exhibited significantly lower inflammation and a trend toward increased pulp regeneration compared to the control group. Histological analysis revealed varying degrees of healing, including reparative dentin formation and calcified tissue. However, pulp necrosis was observed in some cases. While these findings suggest the potential of the gel to improve pulp regeneration, further research with larger sample sizes and human clinical trials is necessary to confirm these results and explore its clinical applications [73].

## 5. Clinical Implications and Future Applications

Early diagnosis and timely management of pulp exposures in primary and immature permanent teeth are critical to maintaining tooth vitality, supporting continued root development, and preserving natural dentition. According to the latest AAPD Clinical Practice Guidelines (2024) [31,32], vital pulp therapy (VPT) procedures—including indirect pulp treatment, direct pulp capping, partial pulpotomy, and full pulpotomy—are appropriate for primary teeth with normal pulp or reversible pulpitis, and for immature permanent teeth with vital pulp, including some cases with signs of irreversible pulpitis.

In primary teeth, pulpotomy remains the most commonly indicated VPT technique when caries removal results in pulp exposure. Full pulpotomy with a bioactive material such as MTA or Biodentine is recommended over formocresol due to superior biocompatibility and clinical success. For immature permanent teeth, partial or full pulpotomy is favored to promote continued root maturation (apexogenesis) when pulp vitality is retained.

For widespread clinical adoption, ideal bioactive materials should offer ease of handling, fast setting times, excellent sealing ability, and high biocompatibility. Recent innovations—such as calcium silicate-based cements, platelet-rich plasma (PRP), and nitric oxide-releasing nanomatrix gels—exemplify materials that not only protect exposed pulp but also actively promote tissue regeneration. These materials enhance healing by releasing bioactive molecules and growth factors that modulate inflammation and stimulate dentinogenesis.

In line with AAPD guidelines, selection of VPT materials and techniques should be guided by pulpal diagnosis, bleeding control, restoration integrity, and the tooth’s strategic value. Integrating regenerative strategies into routine clinical practice can transform the management of pulpal-involved teeth. A shift toward biologically based, minimally invasive therapies prioritize tissue preservation and patient-centered care. Continued advancements in biomaterials and delivery systems will further support this transition, improving long-term treatment success in both pediatric and adult populations.

## 6. Conclusions

Vital pulp therapy (VPT) has evolved significantly, transitioning from the use of calcium hydroxide as a traditional standard to advanced biomaterials such as calcium silicate cements. These materials, alongside improved diagnostic methods like cone-beam computed tomography and molecular biomarkers, have enhanced clinical precision by promoting dentin regeneration, pulp healing, and reducing inflammation.

This review highlights the strengths and limitations of current VPT materials and techniques, emphasizing the need for further research to standardize methodologies and develop innovative biomaterials. Emerging technologies, such as platelet-rich plasma, nitric oxide-releasing nanomatrix gels, and biomimetic scaffolds, hold great promise for advancing pulp preservation and regeneration. By fostering minimally invasive approaches and patient-centered care, VPT is positioned to reduce dependence on invasive procedures like root canals and extractions, offering sustainable, long-term solutions. These advancements underscore the future of VPT as a promotion of regenerative dentistry.

## Supplementary Material

Supplement Figure 1. Simplified flow diagram illustrating the literature selection process for this narrative review.

The following supporting information can be downloaded at: https://www.mdpi.com/article/10.3390/app15094699/s1, Figure S1: Simplified flow diagram illustrating the literature selection process for this narrative review. Although a formal PRISMA diagram is not required for narrative reviews, this figure outlines the identification, screening, eligibility assessment, and inclusion steps followed to ensure transparency in the article selection process.

## Figures and Tables

**Table 1.** An overview of conventional dental biomaterials used in vital pulp therapy.

Biomaterial	Brand Name	Composition	Mechanism of Action	Advantages	Limitations
Calcium Hydroxide [47]	Dycal–Dentsply	Ca(OH)_2_ in paste or powder form	Releases Ca^2+^ and OH^−^ inducing mineralized barrier formation, and antibacterial properties	Biocompatibility promotes hard tissue formation	Low mechanical properties and porosity in the newly formed mineralized tissue Tissue necrosis and inflammation High Solubility
Mineral Trioxide Aggregate (MTA) [51]	ProRoot MTA–Denstply MTA Angelus–Angelus NeoMTA plus–Avalon	Tricalcium silicate, dicalcium silicate, tricalcium aluminate	Promotes dentin bridge formation, seals pulp exposure, and releases Ca^2+^	High biocompatibility, durable seal	Prolonged setting time Lower compressive strength and hardness than TheraCal LC Discoloration potential
Biodentine [52,53]	Biodentine by Septodont	Calcium silicate-based with additives (zirconium oxide, etc.)	Similar to MTA, fast-setting, forms hydroxyapatite	Stimulates odontoblastic differentiation [51] Color Stability [52,53] Low Cytotoxicity [52,53]	Less radiopacity than MTA. Lower wash-out resistance
Endo sequence Root Repair Material [54]	Endosequence–Brasseler	Calcium phosphate, calcium silicate-based	Seals pulp exposure, biocompatible,	No mixing required, high sealing ability, Easy Handling	Limited data on long-term effectiveness, and more expensive
TheraCal LC [55]	Theracal–Bisco	Light-curable calcium silicate	Forms a durable layer, calcium release	Easy handling promotes healing	Low biocompatibility and high cytotoxicity due to the presence of monomers like BisGMA, HEMA, TEGDMA, and UDMA

**Table 2.** An overview of regenerative approaches for VPT.

Biomaterial	Type of Study	Composition	Mechanism of Action	Advantages	Limitation
Collagen, Gelatin, and Gelatin-Methacrylate [59,60]	In Vitro	Collagen-sourced from animal sources Gelatin-collagen denaturation Modified gelatin with methacrylate groups	Influences cellular morphology, differentiation and adhesion	Exhibits low immunogenicity, permeability, porosity, biocompatibility, and biodegradability	Inadequate mechanical strength and structural stability upon hydration
Chitosan [15,61]	In Vitro In Vivo and RCT	Natural protein derived from animals	Mimics dentin matrix protein 1 (DMP1)	Restores the structural integrity of demineralized dentin	Requires extended treatment duration (7–14 days)
			Facilitates both intra-fibrillary (within collagen fibers) and extra-fibrillary remineralization	Biocompatible alternative to natural proteins	Long-term stability under oral conditions needs validation
Alginate [16]	In Vivo	Alginate reinforced Laponite Hydrosphere with hDPCs and VEGF	Promotes extracellular matrix deposition (fibronectin and collagen type I) and vascularized pulp-like tissue formation through VEGF-mediated angiogenesis	Supports hDPSC differentiation, extracellular matrix deposition, and micro-vessel formation	Requires precise control over microsphere size and composition during preparation
				Biocompatibility: High cell viability	Limited Long-Term Data: The study focuses on short-term outcomes (1 month in vivo), with long-term efficacy yet to be validated
Platelet-Rich Plasma (PRP) and Platelet-Rich Fibrin (PRF) [62,63]	Clinical trial	Autologous blood-derived plasma and fibrin clot	Delivers growth factors, promotes pulp healing	Natural, promotes pulp regeneration	Limited availability, patient-specific
Demineralized Dentin Matrix(DDM) [52,64,65]	In Vivo and RCT	Fresh dentin Demineralization using EDTA or hydrochloric acid	Promotes dentinal bridge formation	Stimulates the formation of ordered odontoblast layers and a homogeneous tubular structure	May have variable performance depending on the quality of the dentin matrix source
				Offers a natural alternative to silicate-based cements for healing dentin defects	Long-term clinical efficacy still needs to be established through further studies
Laser-Assisted VPT [51,66]	RCT Fotona–Erbium-doped Yttrium Aluminium Garnet, Biolase –Erbium Chromium laser	Laser technology (e.g., Erbium, Neodymium)	Laser application sterilizes pulp and promotes wound healing; may stimulate dentinogenesis	Minimally invasive, enhances disinfection, promotes healing	Requires specialized equipment, technique-sensitive, high initial cost
Nanohydroxyapatite (nHAp)/NanoMatrix [17,53]	In Vitro In Vivo	Nanoscale hydroxyapatite particles, often in paste or gel Reinforced with Nitric-Oxide peptide	Mimics dentin, promotes mineralization, and dentin bridge formation	Biocompatible, low inflammatory, and proangiogenic response	Limited studies may require longer-term evaluations

## Data Availability

The raw data supporting the conclusions of this article will be made available by the authors upon request.

## Abbreviations

The following abbreviations are used in this manuscript:

AAPD: American Academy of Pediatric Dentistry
VPT: Vital Pulp Therapy
IPT: Indirect Pulp Treatment
DPC: Direct Pulp Capping
CH: Calcium Hydroxide
CSC: Calcium Silicate Cement
GIC: Glass Ionomer Cement
MTA: Mineral Trioxide Aggregate
RMGIC: Resin Modified Glass Ionomer Cement
PRP: Platelet-Rich Plasma
PRF: Platelet-Rich Fibrin
NO: Nitric Oxide
RCTs: Randomized controlled trials
CBCT: cone-beam computed tomography
