# Supplementary material for: Innovations in Bioactive Materials for Dental Pulp Vitality Preservation in Children and Adolescents
Source: Appl Sci (Basel). Author manuscript; Available in PMC 2025 Dec 11. (PMC12692128; doi:10.3390/app15094699)
Supplement: Supplement Figure 1. Simplified flow diagram illustrating the literature selection process for this narrative review. [file NIHMS2124251-supplement-Supplement_Figure_1__Simplified_flow_diagram_illustrating_the_literature_selection_process_for_this_narrative_review_.pdf]

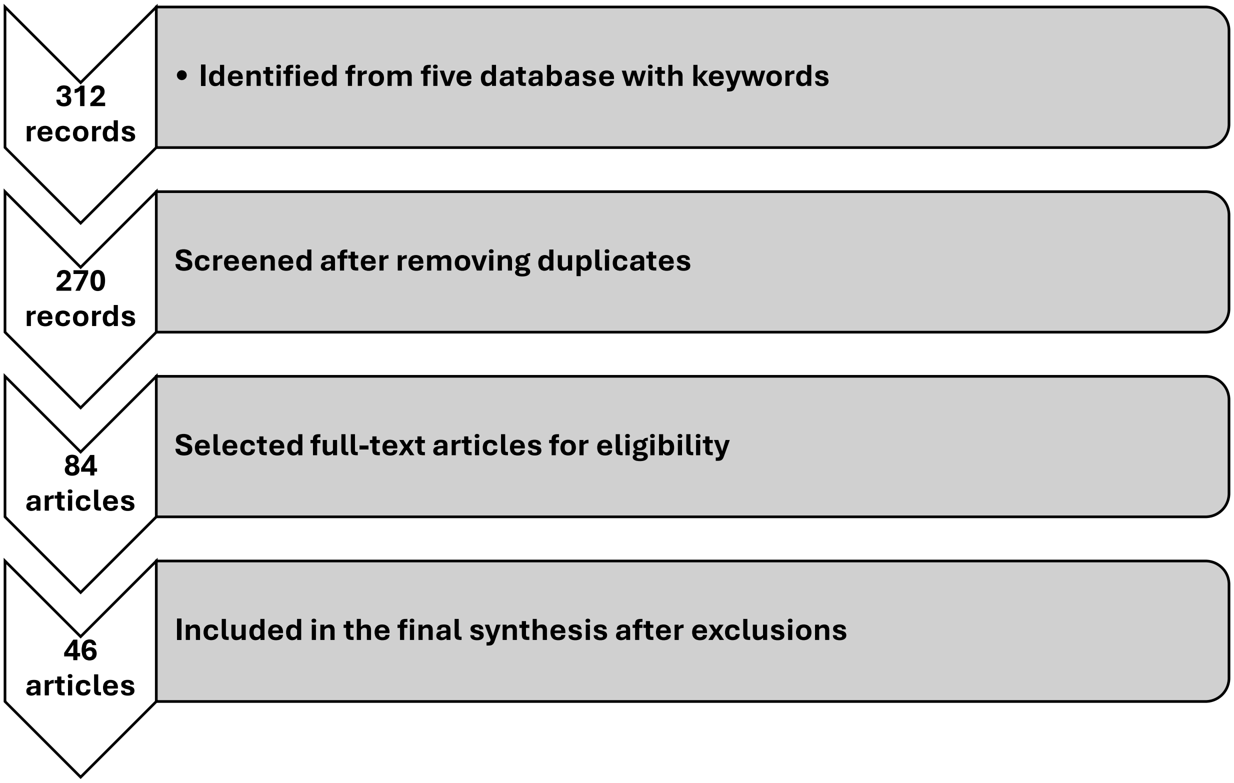

Supplement Figure 1. Simplified flow diagram illustrating the literature selection process for this narrative review. Although a formal PRISMA diagram is not required for narrative reviews, this figure outlines the identification, screening, eligibility assessment, and inclusion steps followed to ensure transparency in the article selection process.
